# The Role of Changes in Extracellular Matrix of Cartilage in the Presence of Inflammation on the Pathology of Osteoarthritis

**DOI:** 10.1155/2013/284873

**Published:** 2013-08-28

**Authors:** Maricela Maldonado, Jin Nam

**Affiliations:** ^1^Department of Bioengineering, University of California, 900 University Avenue, Riverside, CA 92521, USA; ^2^Center for Bioengineering Research, University of California, Riverside, CA 92521, USA

## Abstract

Osteoarthritis (OA) is a degenerative disease that affects various tissues surrounding joints such as articular cartilage, subchondral bone, synovial membrane, and ligaments. No therapy is currently available to completely prevent the initiation or progression of the disease partly due to poor understanding of the mechanisms of the disease pathology. Cartilage is the main tissue afflicted by OA, and chondrocytes, the sole cellular component in the tissue, actively participate in the degeneration process. Multiple factors affect the development and progression of OA including inflammation that is sustained during the progression of the disease and alteration in biomechanical conditions due to wear and tear or trauma in cartilage. During the progression of OA, extracellular matrix (ECM) of cartilage is actively remodeled by chondrocytes under inflammatory conditions. This alteration of ECM, in turn, changes the biomechanical environment of chondrocytes, which further drives the progression of the disease in the presence of inflammation. The changes in ECM composition and structure also prevent participation of mesenchymal stem cells in the repair process by inhibiting their chondrogenic differentiation. This review focuses on how inflammation-induced ECM remodeling disturbs cellular activities to prevent self-regeneration of cartilage in the pathology of OA.

## 1. Introduction

Osteoarthritis (OA) is a debilitating disease, which primarily affects joints, especially load-bearing areas such as hips and knees. It is characterized by pain and degenerative changes in the tissues surrounding those areas. There are no current therapies which can completely prevent the progression of the disease. Some of the main factors that drive the progression of OA are chronic inflammation and gradual structural changes within the joint tissues [1]. Unlike the general concept of OA being a degenerative disease, the remodeling processes are highly active throughout each stage of the disease [2]. During the active remodeling, however, the quality of extracellular matrix (ECM) is compromised due to the quick turnover rate and atypical composition of the newly synthesized ECMs [3]. Among many factors, inflammatory cytokines and proteases are main contributors which mediate the changes in the quality of ECM [2]. As a consequence of the microenvironmental changes, the altered ECM synthesis in the presence of inflammation, in turn, further disturbs the functions of the cells. Therefore, there is a constant cycle of evolution between the cells and their newly synthesized ECM, forming a positive feedback loop, which drives the progression of OA. In this review, we will focus on the interplay between ECM and cellular functions under inflammation, and how these factors are responsible for the progression of OA. An understanding of the complexity of the interplay between the cells and their microenvironment may provide a sound basis for developing suitable therapies to treat osteoarthritis.

## 2. Changes in Extracellular Matrix Synthesis during Osteoarthritis

Progression of OA can be characterized by changes in ECM composition and structure. Natural, healthy cartilage matrix is mainly composed of collagen type II which provides tensile support for the tissue. Aggrecan, a negatively charged proteoglycan that attracts water molecules, provides the compressive resistant and shock absorbing capability of cartilage under loading [2]. It has been shown that during OA, there are sequential events that affect the integrity of homeostatic ECM; aggrecan content is decreased, while collagen content is increased [2, 3, 5]. This change in ECM composition predisposes the tissue for mechanical fault resulting in significantly altered mechanical environments of the cells within the cartilage matrix. 

In the initial stages of OA, proliferative chondrocytes form clusters in order to adjust to the changing microenvironments [2]. This alteration of cellular configuration also changes the quantity and composition of the ECM secreted by the cells. It has been shown that there is a significant downregulation of aggrecan gene expression at the onset of OA in a rat model [1], and this finding agrees with markedly low proteoglycan synthesis, observed in human OA samples with normal appearance [6]. The changes of aggrecan, which exists in a nonaggregated form in OA, alter the permeability and thus mechanical compliance of the matrix [2, 7]. The reduced proteoglycan content decreases compressive modulus of cartilage and consequently exposes the tissue to greater strains when exposed to mechanical stress.

**Figure 1:**
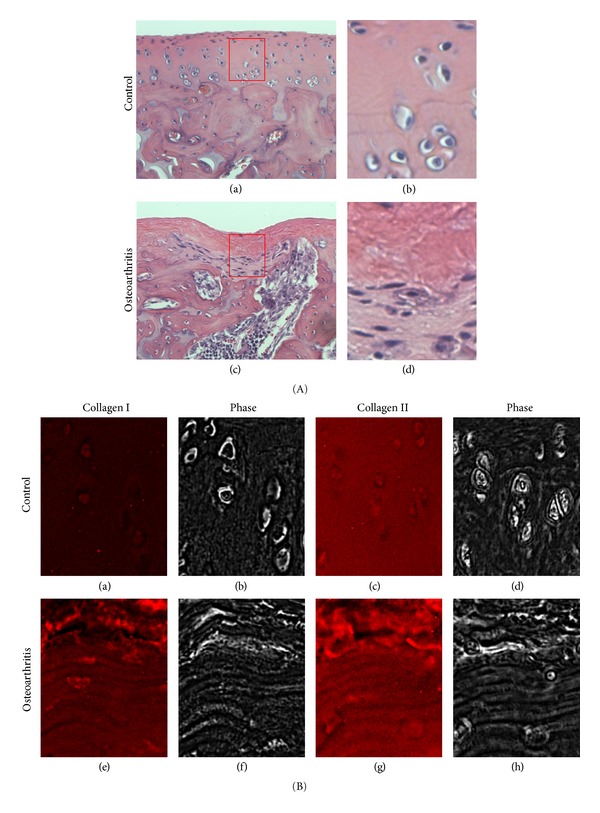
Changes in the extracellular matrix structure and composition of cartilage afflicted by osteoarthritis (OA). Experimental OA was induced by intra-articular injection of monoiodoacetate (MIA) similar to the previously described protocol using a rat model [4]. OA induced rats were sacrificed at day 11, and the medial condyles of the arthritic knees (A (c-d); B (e–h)) were histologically (H&E staining (A)) and immunohistologically (collagen type I (B (a) and (e)) and type II (Figure B (c) and (g)) compared to that of the saline-injected sham control ((A (a-b); B (a–d)). (A) Microscopic features of OA cartilage (grade 2-3) show cartilage lesion formation, articular surface fissurization, subchondral bone advancement, and bone marrow edema/cyst. In addition, cell clustering and fibrocartilage formation is apparent in OA samples. (A) (b and d) are magnified images of the area indicated in (A) (a and c), respectively, to reveal the changes in cellular morphology. (B) Consecutive sections of the healthy and OA cartilages were stained using monoclonal antibodies for collagen type I or type II. An increase in intensity for collagen type I is observed in the OA cartilage, while it is not present in the control cartilage. Collagen type II is readily observed for both the healthy and OA cartilage. (B) (b, d, f, and h) are phase-contrast images of (B) (a, c, e, and g), respectively, to reveal tissue morphologies.

Unlike the decreased production of proteoglycan, collagen synthesis rate increases in the early stages of OA and remains elevated [8]. In addition to the increased ratio of collagen/aggrecan synthesis, the composition of collagen type has been also shown to change from collagen type II to type I [9]. Healthy cartilage matrix mainly contains collagen type II, while collagen type I is mainly found in subchondral bone tissue [2, 3, 10]. The compositional change affects the mechanical stability of the ECM network [10]. Compared to collagen type I, type II chains contain a higher content of hydroxylysine as well as glucosyl and galactosyl residues which mediate the interaction with proteoglycans [11]. Therefore, the decreased collagen type II content during OA inevitably undermines the integrity of ECM networks formed by collagen and proteoglycan. Furthermore, Silver et al. showed that the elastic modulus, due to shortened collagen fibril lengths, decreases with an increased extent of OA [12]. As a result of these changes, the osteoarthritic cartilaginous tissues exhibit a reduced ability to store elastic energy, and this, in turn, leads to fibrillation and fissure formation [12].  Figure 1 shows the structural and compositional changes in cartilage in a monoiodoacetate- (MIA-) induced arthritis model in rats. Although the animal model induces significantly accelerated cartilage degeneration as compared to typical human osteoarthritis, it depicts similar structural and compositional changes in cartilage exhibited in the pathogenesis of OA [13]. On day 11 post-MIA injection, the overall cartilage damage was assessed at Grade 2-3 according to Osteoarthritis Research Society International's (OARSI's) histopathology grading system showing cartilage lesion formation, articular surface fissurization, subchondral bone advancement, and bone marrow edema/cyst [14]. An area exhibiting chondrocyte disorientation without vertical fissure development was chosen to observe changes in cartilage matrix. In this area, nonchondrocytic collagen type I is present in the cartilage matrix of the OA tissue, whereas it is negligible in the control (Figure 1(B)). These changes in the structure and composition of ECM progressively alter the biological and mechanical microenvironments that significantly modulate cellular activities as described later in this review. 

## 3. Inflammation-Induced Extracellular Matrix Changes in Osteoarthritis

ECM changes in cartilage can be attributed to multiple factors during the progression of OA. Among them, inflammation plays an active role affecting both quantity and quality of ECMs. Mechanical damage and/or age-related wear/tear are thought to trigger systematic inflammatory responses in all tissues surrounding the joint including articular cartilage, synovial membrane, subchondral bone, and ligaments [2, 15]. Chondrocytes, the only cell type residing in cartilage, respond to such inflammatory conditions and participate in the catabolic activities that ultimately lead to the degradation of cartilaginous ECM [16]. An animal model of MIA-induced arthritis showed that the sequential upregulation of inflammatory genes is associated with all levels of cartilage damage throughout the progression of OA [1]. These upregulated inflammatory genes form a positive feedback loop, mainly through the NF-*κ*B signaling pathway, as the severity of the cartilage damage progresses [17]. In fact, it was observed that chondrocytes in human arthritic cartilages also constitutively exhibit elevated activities of NF-*κ*B [18]. Factors that contribute to the catabolic processes in OA include interleukin 1*β* (IL-1*β*), tissue necrosis factor-*α* (TNF-*α*), IL-12, IL-15, and various associated chemokines [19–23]. These inflammatory factors were shown to significantly increase the expression of matrix degrading proteins including matrix metalloproteinases (MMPs) (i.e., MMP-1 and MMP-13) and various types of a disintegrin and metalloproteinase with a thrombospondin type 1 motif (ADAMTS) (i.e., ADAMTS 1,4,5) in chondrocytes [1, 24–30]. For example, an increase in cell clustering, a typical morphological feature of chondrocytes in the early stage of OA, was observed with an increase in MMP-13 expression [31]. The receptor for advanced glycation end products (RAGE), which is increased in OA articular chondrocytes, was also shown to stimulate MAP kinase and NF-*κ*B activities that, in turn, increased the production of MMP-13 and propagated the catabolism of the cartilage matrix [32, 33]. 

The degenerative activities of matrix degrading proteins are intensified by the elevated level of nitric oxide (NO), a molecule which is also upregulated by inflammatory proteins in chondrocytes. NO, upregulated by the transcriptional activity of NF-*κ*B, perpetuates the chronic inflammation that enhances matrix degradation and mediates apoptosis of chondrocytes by creating oxidative environments [34–36]. In a canine model of OA, the use of a NO inhibitor reduced the degenerative changes in cartilage, possibly demonstrating the critical role of NO in the progression of OA [34]. 

Concurrently with matrix degradation, the inflammation-mediated downregulation of chondrogenic growth/transcription factors that mediate chondrocytic ECM synthesis, such as transforming growth factor *β* (TGF-*β*), sex determining region Y-box 9 (SOX9), insulin-like growth factor (IGF), and connective tissue growth factor (CTGF), is also responsible for suppressing the anabolic activities of chondrocytes [1, 37, 38]. Taken together, these results demonstrate the significant influence of inflammatory mediators in the progression of OA by altering the homeostasis of cartilage ECM.

Another matrix component which is found in increased concentrations in synovial fluid during OA is Tenascin-C (TN-C), an ECM glycoprotein. Elevated levels of TN-C have been suggested to induce inflammatory mediators and promote ECM degradation in OA patients [39]. Although TN-C is highly expressed during embryogenesis, its presence is minimal in healthy adult tissues. Its expression during OA is, however, highly upregulated [40, 41]. The elevated concentration of TN-C causes a significant effect in the catabolism of the cartilage, resulting in degradation of ECM [39, 40]. Additionally, biglycan fragments in articular cartilage and meniscus and fibronectin fragments in hip and knee synovia have also been found in elevated levels as OA progresses [42–44]. Both fragmented biglycan and fibronectin exhibit proinflammatory effects through the activation of toll-like receptors [45, 46]. Overall, the combination of inflammation-induced upregulation of matrix-degrading proteins, downregulation of chondrocytic ECM synthesis, and accelerated matrix degradation due to fragmented inflammatory ECMs, promotes the progression of disease.

## 4. Alteration in Biomechanical Environments during Osteoarthritis

The changes in altered ECM synthesis and elevated activities of matrix degrading proteins drastically change the mechanical properties of cartilage, which further intensifies the destructive processes associated with OA [47]. Initially, an increase in cartilage thickness is observed by hyperproliferative chondrocytes before noticeable surface fibrillation occurs [48]. The highly proliferating chondrocytes produce greater amount of aggrecan that leads to cartilage thickening in dimensions as well as softening of extracellular matrix [2]. At this stage, a lower shear modulus was observed in the cartilage from an OA model when compared to normal articular cartilage [49, 50]. In a mouse model, a reduction in tensile stiffness in articular cartilage is also accompanied by the tentative cartilage thickening [51]. These biomechanical changes expose chondrocytes to an environment more susceptible to greater strains, as compared to physiological levels, thus altering their cellular functions. 

As the disease progresses, however, the tissue gradually loses aggrecan content, which has provided compliance of local mechanical environments due to its ability to interact with water molecules. In addition to aggrecan loss, it has been recently shown that collagen fibril stiffens in osteoarthritic cartilage [52]. Furthermore, another possible mechanism through which the mechanical microenvironment changes is the accumulation of advanced glycation end products (AGEs) which can crosslink to the collagen network [53]. In vitro, the increased AGE crosslinking to the collagen network was shown to increase the stiffness of human adult articular cartilage [53]. The combination of aggrecan loss and collagen network stiffening results in increased overall stiffness of the tissue. Consequently, as OA advances, the cartilage layer becomes thinner and stiffer transmitting greater load to the underlying subchondral bones. The change in mechanical conditions induces the advancement of subchondral bones towards the articular surface leading to the development of bone marrow edema/subchondral bone cysts and the propagation of periarticular osteophytes [2, 54, 55]. Recent studies suggest that these changes in subchondral bone structure may precede the articular cartilage thinning [56]. 

Nevertheless, due to changes in the mechanical properties of the cartilage via altered homeostasis of ECM, its residing cells, chondrocytes, are exposed to vastly different biomechanical microenvironments that further intensify the progression of OA by altering cellular behaviors. Ultimately, this leads to the formation of fibrocartilaginous tissues that exhibit more bone-like properties replacing the completely degenerated cartilage in addition to osteophyte formation at the periphery of the articular surface [2, 54]. 

## 5. The Effects of Inflammation on Cartilage Extracellular Matrix Homeostasis by Articular Chondrocytes

Global inflammation in synovium during OA affects chondrocytes that are responsible for ECM turnover and thus cartilage homeostasis [57]. Inflammation which is persistent in OA has shown to directly induce the catabolic activities of chondrocytes. IL-1*β*, a highly upregulated cytokine during OA, has shown to induce upregulation of matrix degrading enzymes such as MMP-1, 3, and 13 in chondrocytes [58]. Dozin et al. also showed that when exposed to inflammatory cytokines, chondrocytes, regardless of patient age or OA status of human donors, enhance their production of proinflammatory cytokines such as IL-6 and IL-8 [59]. TNF-*α*, another critical cytokine that is highly upregulated in OA, has been shown to induce MMP-13 expression, mediated by ERK, p38, JNK MAP kinases, and AP-1 and NF-*κ*B transcriptions factors [24, 60, 61]. At the same time, the presence of inflammatory cytokine IL-1*β* has been shown to play a role in suppressed ECM synthesis through downregulation of SOX9 [62]. This, in turn, decreases the expression of collagen type II and aggrecan in articular chondrocytes. The activation/suppression of such signaling cascades autoregulates chondrocytes to further upregulate the synthesis of matrix degrading enzymes and downregulate the production of chondrocytic ECMs [63]. Nitric oxide (NO) and cyclooxygenase-2 (COX-2), two components which have active roles in perpetuating inflammation, were also endogenously expressed at high levels in chondrocytes from OA tissues even when cultured in vitro in the absence of inflammatory cytokines [64, 65]. These changes in metabolism may demonstrate a possible permanent phenotypical change in the OA chondrocytes.

In this regard, one notable alteration of chondrocytes in arthritic joints is their production of nonchondrocytic ECM. In addition to the increase in the production of collagen type I replacing type II as previously described, chondrocytes isolated from OA diseased tissues have shown to produce collagen type X, a marker for hypertrophic chondrocytes, as compared to undetectable expression of the protein in healthy cartilage [66]. Collagen type X is typically synthesized by hypertrophic chondrocytes that also produce collagen type I. The emergence of these nonarticular chondrocytic proteins may indicate the change of phenotype in chondrocytes as the disease progresses. The morphological change of chondrocytes with abnormal nonround morphology in arthritic cartilages could be related to a phenotypical change such as an increase in IL-1*β* production and a decrease in pericellular collagen type VI synthesis [67]. When the cells from arthritic knees are subject to a chondrogenic in vitro culture condition, they are not able to fully recover normal tissue phenotype as evident by low cellularity and decreased chondrocytic ECM production as compared to chondrocytes from healthy joints [66, 67]. This demonstrates that damages in OA cartilage may not be able to be fully recovered by autologous chondrocytes.

One possible cause of the phenotype change of OA chondrocytes is inflammation as inflammatory synovial fluid has shown to activate chondrocytes and dramatically affect the normal processes of the cells. When healthy chondrocytes are subjected to inflammation, simulated by inflammatory cytokines such as IL-1*β*, TNF-*α*, CXCL1, or 8, all of which are upregulated during OA, the cells exhibit hypertrophic differentiation [68]. This differentiation is shown to be mediated by RAGE signaling through the p38 MAPK pathway [69]. Interestingly, the activation of the p38 MAPK signaling pathway has also shown to promote the synthesis of MMP-13 possibly linking the change in phenotype to the facilitated rate of matrix turnover [32]. In addition to the synthesis of nonchondrocytic ECM and enhancement in matrix degradation, chronic inflammation also induces cell death. When healthy chondrocytes were subject to synovial fluids from osteoarthritic patients, the cells not only upregulated the expression of cytokines, such as IL-6, IL-8, monocyte chemotactic protein-1 (MCP-1), and vascular endothelial growth factor (VEGF), but also underwent apoptosis [16]. 

## 6. The Effects of Changes in Extracellular Matrix on Articular Chondrocytes

The altered microenvironments by ECM changes, in the presence of inflammation, further drive catabolic/nonreparative activities of chondrocytes, ultimately leading to cartilage destruction/achondrocytic ECM formation. As previously described, the mechanical properties of cartilage are dynamically altered during the progression of OA due to imbalanced matrix turnover (greater matrix degradation versus synthesis) and noncartilaginous ECM formation. The increase in local matrix stiffness due to changes in ECM appears to suppress chondrocytic activities of the cells. Recent studies show that chondrocytes sense the stiffness of the matrix and differentially respond to it by altering their phenotype, resulting in production of different types of ECM (i.e., ratio of collagen type II to type I) [70–72]. An optimal stiffness has been shown to promote greater SOX9, COL2A1, and aggrecan gene expression in chondrocytes and either above or below this stiffness induced dedifferentiation of the cells towards fibrochondrocytic phenotype [70]. This effect of matrix stiffness on modulating chondrogenic phenotype has been shown to occur through the regulation of the TGF-*β* signaling pathway [70]. In addition, the mechanosensitive behavior of chondrocytes may explain the fact that typical in vitro 2D culture of chondrocytes on stiff tissue culture plastics results in the dedifferentiation of the cells [73–75].

The changes in matrix composition during OA not only affect the mechanical environments of chondrocytes but also alter interactions of matrix proteins with the cells. Matrilin-3 (MATN3) is a matrix protein that is highly upregulated during OA [76, 77]. Although the protein is a part of healthy cartilage matrix, the soluble form of MATN3 is upregulated and released to synovial fluid in OA [78]. When human chondrocytes were cultured in the presence of soluble MATN3, there was a decrease in ECM anabolism and increased catabolism only at concentrations higher than those found in OA patients. On the other hand, when soluble MATN3 was immobilized, ECM synthesis and accumulation was enhanced [78]. These results show how MATN3, which is found in synovial fluid of OA patients, can change the behavior of chondrocytes, demonstrating the direct involvement of ECM in the progression of OA by interacting with the cells as well as indirectly by changing the cells' mechanical environments. 

The presence of calcium crystals in cartilage has been shown to increase with severity of OA, and these changes have a strong correlation with hypertrophic chondrocyte differentiation [79]. Interestingly, bovine articular chondrocytes within cartilage explants, when exposed to basic calcium phosphate crystals, had significant increases in intracellular calcium content, which is correlated with cartilage matrix degradation [80]. Another ECM component that affects chondrocyte metabolism is fibronectin, which showed a significant positive correlation between chondrocyte apoptosis and fibronectin content [81]. Overall, these multifaceted effects by changes in ECM, including dysregulation of matrix synthesis (reduction in collagen type II and aggrecan, increase in collagen type I and X), upregulation of matrix degradation, and induction of cell apoptosis, promote the progression of OA by altering the cellular behaviors of chondrocytes. 

## 7. The Effects of Inflammation on Chondrogenic Differentiation of Mesenchymal Stem Cells during Osteoarthritis

The mechanisms involving the initiation of OA are still elusive as some argue it is mechanical damage-induced and others inflammation-induced. Nevertheless, once the disease is initiated, the degeneration of cartilage matrix progresses due to the combination of chronic inflammation and altered mechanical loading as discussed earlier. A part of the progressive degenerative processes is due to the limited regenerative capability of chondrocytes. These cells are typically quiescent in healthy cartilage [2]. When they are exposed to proliferating conditions to repair the cartilage damage, they often dedifferentiate to a phenotype that produces nonchondrocytic ECM [2]. This atypical ECM synthesis further drives chondrocyte dedifferentiation and nonhomeostatic ECM synthesis by altered mechanical environments. In addition to chondrocytes, the repair of the damaged tissue is attempted by another cell type, mesenchymal stem cell (MSC), that can differentiate to all mesenchymal lineage cells including chondrocyte, osteoblast, and adipocyte [82]. MSCs often participate in the repair of bone damage since they constitute bone marrow. Due to its close proximity to the cartilage layer in the subchondral marrow and their ability to differentiate into chondrocytes, MSCs have been considered as a possible cell source involved in cartilage repair.

For this reason, microfracture (or microperforation) surgery is often used to treat a localized cartilage lesion. Small fractures are created in the subchondral bone, and this causes new cartilage formation mainly due to the regenerative activities of MSCs from the bone marrow [83]. Although this technique has shown some benefits repairing damaged cartilage, the neotissue contains fibrocartilage that exhibits different mechanical properties, leading to question its long-term stability [84, 85]. These studies may provide clues for why endogenous MSCs cannot fully rescue damaged cartilage during the progression of osteoarthritis, unlike the positive healing response after bone fractures. Typically, subchondral bone advances towards the cartilage surface as the articular surface degrades [86]. In this condition, MSCs are subjected to a milieu of inflammation, altered ECM composition, and vastly different mechanical loading profiles in the injured cartilage, all of which affect the differentiation of MSCs to chondrocytes.

As described earlier, the native cartilage is exposed to chronic inflammation conditions by increased levels of inflammatory mediators including IL-1*β*, TNF-*α*, and prostaglandin E_2_ (PGE_2_) [87, 88]. These inflammatory cytokines not only affect the homeostatic functions of residential chondrocytes but also impact the chondrogenic differentiation of MSCs [87, 89–91]. Treatment of IL-1*β* during chondrogenic differentiation of bone marrow-derived MSCs suppresses Sox9 expression, a critical transcription factor that controls chondrogenesis [90]. The suppression of Sox9 subsequently leads to a decrease in collagen type II and aggrecan expression. In addition, TNF-*α*, in combination with IL-1*β*, has been shown to transform embryonic chondroprogenitor cells into fibroblast-like cells, further suggesting the inhibitory effects of inflammatory cytokines on chondrogenesis [87]. Similarly, when human MSCs are exposed to conditioned medium derived from osteoarthritic synovium, chondrogenesis is inhibited [92]. These antichondrogenic effects of inflammatory cytokines were shown to be caused by the activation of the NF-*κ*B signaling pathway [93]. Overall, inflammatory conditions present in OA cartilage prevent chondrocytic differentiation of MSCs, thus inhibiting regeneration of damaged cartilage with appropriate chondrocytic ECMs.

## 8. The Effects of Changes in Extracellular Matrix on Chondrocytic Differentiation of Mesenchymal Stem Cell

The changes in the composition of ECM also affect chondrogenic differentiation of MSCs. In a study by Bosnakovski et al., MSCs cultured in collagen type II hydrogels exhibited greater gene expression levels of chondrocytic markers as compared to those cultured on typical tissue culture plates [94]. As OA progresses, residential chondrocytes start to produce collagen type I instead of type II. This change can affect the subsequent chondrogenesis of MSCs as it has been shown that collagen type II favors chondrogenic induction by modulating cell shape, as compared to collagen type I [95]. It was demonstrated that collagen type II promotes a more rounded cell shape, similar to that of the native chondrocyte in healthy cartilage, through the *β*1 integrin-mediated Rho A/Rock signaling pathway.

In addition to the compositional effect, mechanical changes of ECMs (become stiffer due to the loss of hydrating aggrecan in OA) affect chondrogenesis of MSCs by regulating cell morphology [96]. A softer mechanical environment enhances chondrogenesis of MSCs, evident by greater gene and protein expression of chondrogenic markers including SOX9, collagen type II, and aggrecan by inhibiting stress fiber formation, as compared to the stiffer environment. Similarly, using polyacrylamide hydrogels with varying stiffnesses, Xue et al. showed that human mesenchymal stem cells are differentiated towards a chondrocytic phenotype on softer gels, regardless of initial cell seeding density [97]. The study highlights the importance of cell-matrix interactions during chondrogenic differentiation of MSCs. 

Along with the direct influence of local stiffness change on MSC differentiation, the altered mechanical profiles under loading also affect the differentiation process. Bone marrow derived MSCs seeded onto fibrin hydrogels developed a spread out morphology and differentiated towards a myogenic lineage [98]. In the presence of long-term, dynamic compression, myogenic differentiation was inhibited, while markers for chondrogenic phenotype were upregulated. However, the magnitude of loading is an important factor determining chondrocytic differentiation of MSCs and thus synthesis of proper ECMs. Under the same loading regimen, a stiffer ECM induces less strain on the cells. In this regard, Michalopoulos et al. have recently shown that physiological compressive loading (15% strain) on MSC-laden scaffolds induces greater chondrogenesis as compared to a smaller strain of 10% that led to greater osteogenesis [99]. Similarly, stiffer agarose gels inhibited cartilage matrix production and gene expression of MSCs under hydrostatic pressure as compared to those in softer microenvironments [100]. These studies demonstrate that changes in the mechanical properties of cartilage during OA may favor the differentiation of MSCs towards nonchondrocytic lineages further intensifying the degeneration of cartilage. Overall, altered environments in ECM composition and mechanical properties during the progression of OA significantly limit the chondrogenesis of MSCs inhibiting the regeneration process of cartilage damage.

## 9. Summary

**Figure 2:**
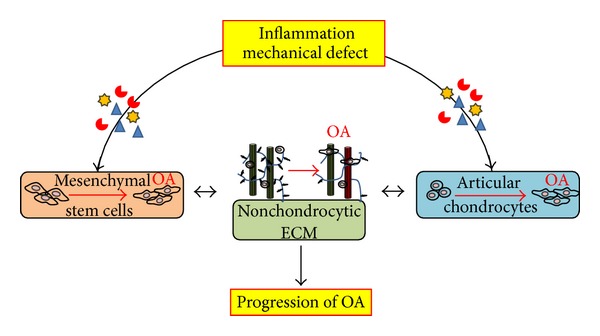
Schematic of the interplay between the extracellular matrix and cellular activities under inflammation during the progression of osteoarthritis (OA). Wear and tear or trauma induces inflammation and mechanical defects in cartilage, which initiate OA. These altered microenvironments affect the residential chondrocytes to produce nonchondrocytic extracellular matrix (ECM) that, in turn, further drives the dedifferentiation of the chondrocytes. The changes in microenvironments also negatively affect the chondrogenic differentiation of mesenchymal stem cells that originate from subchondral bone marrow, preventing the self-regeneration of cartilage. The positive feedback loop between mal-formed ECM and cellular activities drives the progression of OA.

Both inflammatory factors and compositional/structural changes of ECM drive the progression of OA by affecting residential articular chondrocytes as well as MSCs that migrate from bone marrow in the underlying subchondral bone to repair the cartilage defect (Figure 2). Due to chronic inflammation and altered microenvironments, chondrocytes change their phenotype towards more hypertrophic cells resulting in achondrocytic ECM synthesis. These changes in ECM, in combination with cartilage matrix degradation under inflammation, further fuel the degeneration process resulting in the alteration of biomechanical conditions, which disturb the surrounding tissues in the joint. The ECM changes in the presence of inflammation also negatively affect chondrogenic differentiation of MSCs, limiting self-regeneration of cartilage. Overall, the interplay between changes in ECM and changes in cellular function under inflammation forms a positive feedback loop that drives the pathology of OA.
